# Peroxisome Proliferator-Activated Receptor Family of Lipid-Activated Nuclear Receptors Alpha Silencing Promotes Oxidative Stress and Hypertrophic Phenotype in Rat Cardiac Cells

**DOI:** 10.3390/antiox14091059

**Published:** 2025-08-28

**Authors:** Marzia Bianchi, Nadia Panera, Sara Petrillo, Nicolò Cicolani, Cristiano De Stefanis, Marco Scarsella, Domenico Ciavardelli, Fiorella Piemonte, Anna Alisi, Anna Pastore

**Affiliations:** 1 Research Unit of Genetics of Complex Phenotypes, Bambino Gesù Children’s Hospital, IRCCS, 00146 Rome, Italy; marzia.bianchi@opbg.net (M.B.); nadia.panera@opbg.net (N.P.); anna.pastore@opbg.net (A.P.); 2 Unit of Muscular and Neurodegenerative Diseases, Bambino Gesù Children’s Hospital, IRCCS, 00165 Rome, Italy; sara.petrillo@opbg.net (S.P.); fiorella.piemonte@opbg.net (F.P.); 3 Core Facilities, Bambino Gesù Children’s Hospital, IRCCS, 00146 Rome, Italy; nicolo.cicolani@opbg.net (N.C.); cristiano.destefanis@opbg.net (C.D.S.); marco.scarsella@opbg.net (M.S.); 4 School of Medicine and Surgery, University “Kore” of Enna, 94100 Enna, Italy; domenico.ciavardelli@unikore.it

**Keywords:** PPARα, ROS, Ebselen, cardiac hypertrophy

## Abstract

The peroxisome proliferator-activated receptor family of lipid-activated nuclear receptors (PPARs) plays a critical role in the regulation of cellular lipid metabolism. In cardiac muscle, PPARα is highly expressed and regulates genes involved in fatty acid oxidation, but its activity is downregulated in hypertrophic hearts; however, the consequences of chronic PPARα deficiency on the cardiac contractile apparatus remain unclear. This study aimed to investigate the PPARα role in hypertrophic phenotype and to evaluate the potential effects of the antioxidant Ebselen (Ebs) treatment on changes associated with PPARα depletion. We thus generated an in vitro model of cardiac hypertrophy by stable silencing of the *PPARA* gene in H9c2 rat cardiomyoblasts. We observed that PPARα silencing induces a hypertrophic phenotype, characterized by increased *NPPB* and decreased *FBXO32* expression, mitochondrial dysregulation, impaired lipid metabolism, oxidative stress, and ferroptosis-related alterations. Epigenetically, H3K27ac levels increased while H3K27me3 decreased. Moreover, miR-34a, miR-132, and miR-331 were downregulated, implicating a miRNA-mediated mechanism in PPARα-linked cardiac hypertrophy. Treatment with Ebs, a redox-active compound with inhibitory effects on ferroptosis and epigenetics, reversed hypertrophic phenotype and restored miRNA levels. In conclusion, we found that PPARα depletion promotes oxidative stress and hypertrophic phenotype and that Ebs may act as a potential therapeutic agent.

## 1. Introduction

The primary function of the heart is to preserve the perfusion of peripheral organs, following their demand during both normal and stress conditions. After birth, cardiomyocytes rapidly become terminally differentiated, so in adulthood there is no increase in cardiomyocyte number, but a compensatory effect expanding individual cardiomyocyte size [1], a process termed hypertrophy. There are two types of hypertrophy: physiological and pathological. The physiological one is an adaptive response to reduce ventricular wall stress, preserving function and efficiency in response to an increased workload demand; in response to pathological stimuli, hypertrophy generally progresses to ventricular chamber dilatation with wall thinning, to heart failure, to arrhythmias, and death [1,2]. Different cellular signaling pathways, such as cellular metabolism and proliferation, epigenetic modifications, and oxidative stress stimuli [1], modulate cardiac hypertrophy (CH).

The peroxisome proliferator-activated receptor (PPAR) family is a group of three nuclear receptors, including PPARα, β/δ, and γ, that play a pivotal role in maintaining energy homeostasis by acting as lipid-sensing transcription factors [3]. Cardiac muscle expresses relatively high levels of PPARα, where it activates the transcription of several genes involved in fatty acid uptake and oxidation [3]. Noteworthy in vitro and in vivo models of cardiac hypertrophy showed a decreased expression of the gene encoding for PPARα (*PPARA*) and reduced biochemical activity [4]. This modulation implies a metabolic change that has not yet been understood. Indeed, it is unclear whether the shift from fatty acid oxidation to glucose metabolism has to be considered as a defensive mechanism that preserves heart contractility or an initial stage of cardiac damage [4]. The functional role of PPARα in cardiac muscle has been investigated in an in vivo murine model with *PPARA* gene deficiency [5,6]. In gain-of-function transgenic mouse models, the increase in PPARα activity and the linked rise in fatty acid oxidation caused an increase in reactive oxygen intermediates [7]. On the other hand, PPARα activation was reported as a crucial event to prevent cellular oxidative damage resulting from metabolism, oxidative stress, and inflammation. In particular, the latter effect may be achieved by the PPARα-dependent repression of nuclear factor-κB (NF-κB) signaling and the consequent reduction in pro-inflammatory cytokine production [8]. The protective role of PPARα was also confirmed by studies on *PPARA*-deficient mice [5,6]. Despite a normal life span, these models exhibited cardiac fibrosis characterized by myocardial damage [5] and atypical mitochondrial morphology [6]. Therefore, chronic deactivation of the PPARα signaling pathway may disrupt the normal equilibrium between oxidant and antioxidant molecules, contributing to cardiac damage [5]. Despite these lines of evidence, PPARα-dependent processes in (CH) remain fragmentary and incomplete, and the exact consequences of chronic PPARα depletion on cardiac muscle contractility and performance require further investigation.

The aim of this study was thus to investigate the exact role of PPARα in hypertrophic phenotype in an in vitro model of CH by using the H9c2 cardiomyoblasts derived from rat ventricles, which represent a well-characterized reliable model for cardiovascular research [9], by the stable silencing of *PPARA* gene expression by the short hairpin RNA (shRNA) approach. In order to clarify the nature of PPARα-dependent processes in CH, we investigated cell morphology, the expression of major hypertrophic and ferroptosis genes, and antioxidant response in growing basal conditions, exploring possible mechanisms. Finally, we tested the possible drug-induced rescue of a normal phenotype by treating cells with the antioxidant and anti-ferroptosis molecule Ebselen (Ebs) [10,11].

## 2. Materials and Methods

### 2.1. Cell Cultures and Treatments

The H9c2 rat cardiomyoblasts were obtained from American Type Cell Collection (ATCC, Manassas, VA, USA). Cells were cultured in Dulbecco’s modified Eagle’s medium (Euroclone, Whetherby, UK) and supplemented with 10% *w*/*v* fetal bovine serum (Euroclone, Whetherby, UK), 100 mg/mL streptomycin, and 100 U/mL of penicillin (Euroclone, Whetherby, UK). During all the experiments and during expansion, cells were kept at 37 °C in a 95% air and 5% carbon dioxide humidified atmosphere. The cells were sub-cultured at a 1:2 ratio to prevent differentiation; cells were split before reaching 70% confluence. During logarithmic growth, the cells were treated with Phosphate Buffered Saline (PBS) or 50 μmol/L H_2_O_2_ (Sigma-Aldrich, St. Louis, MO, USA) for 1 h and harvested after 5 days after H_2_O_2_ removal. Ebs (Sigma-Aldrich, St. Louis, MO, USA) or PBS, as a vehicle, was added to the growth medium at 5 μmol/L concentration for 36 h.

### 2.2. PPARA Stable Silencing

Cells were plated the day before the transfection at 80% confluence in 35 mm dishes. *PPARA* stable depletion was performed by ready-to-use short hairpin (shRNA) retroviral plasmids purchased from Origene (OriGene Technologies, Inc., Rockville, MD, USA). In detail, 250 ng of plasmids were transfected using Lipofectamine 2000 (Thermo Fischer Scientific, Waltham, MA, USA) following the manufacturer’s instructions. The following shRNA plasmids were used: 29-mer scrambled shRNA cassette in pGFP-V-RS (Scramble, TR30013, OriGene Technologies, Inc., Rockville, MD, USA) as control, or 4 unique 29mer shRNA constructs in retroviral GFP vector for rat PPARα shRNAs (Gene ID: 25747, TG7097836A-D, OriGene Technologies, Inc., Rockville, MD, USA). We obtained stably transfected clones by using antibiotic selection 0.5 μg/mL of puromycin (Sigma-Aldrich, St. Louis, MO, USA) in the culture medium for two weeks.

### 2.3. Quantitative Real-Time PCR (qRT-PCR)

Total RNA was isolated from 100 mm cell culture dishes using the Total RNA Purification Plus Kit (Norgen BioTek Corp., Thorold, ON, Canada) according to the manufacturer’s protocol. cDNA was synthesized using the SensiFast cDNA Synthesis Kit (Meridian Bioscience, Milan, Italy) according to the kit’s instructions. The quantitative gene expression was performed by using 15 ng cDNAs in a 10 μL reaction volume containing the following: 2× TaqMan Universal PCR Master Mix, No AmpErase UNG (Thermo Fischer Scientific, Waltham, MA, USA), and qPCR probe-based assays (Thermo Fischer Scientific, Waltham, MA, USA), including *PPARA* (Rn00566193_m1); Acyl-CoA Oxidase 1 (*ACOX1*) (Rn01460628_m1); Muscle Atrophy F-box gene (*FBXO32*) (Rn00591730_m1); Forkhead box O3 gene (*FOXO3*) (Rn01441087_m1); Nuclear factor erythroid 2 gene (*NFE2*) (Rn01533343_m1); Brain Natriuretic Peptide B gene (*NPPB*) (Rn00580641_m1) and Glyceraldehyde-3-Phosphate Dehydrogenase gene (*GAPDH*) (Rn01775763_g1) as housekeeping; or 2× SYBR Green PCR Master Mix (Thermo Fischer Scientific, Waltham, MA, USA), along with primers obtained from Sigma-Aldrich (St. Louis, MO, USA) and listed in Supplementary Table S1.

The detection of microRNAs (miRNAs) was performed by qRT-PCR using 2× SYBR Green PCR Master Mix (Thermo Fischer Scientific, Waltham, MA, USA) and primers purchased from Sigma-Aldrich (St. Louis, MO, USA) and listed in Supplementary Table S1.

Briefly, cDNA was synthesized using the miRCURY LNA RT Kit (#339340, Qiagen, Hilden, Germany) according to the manufacturer’s instructions. An amount of 10 ng of total RNA, in a 10 μL-RT reaction, was used as starting material, and the procedure involved incubating the reaction at 40–42 °C for 1 h, followed by an inactivation step through a brief incubation at 95 °C.

The cDNAs were amplified in the QuantStudio™ 7Pro Real-Time PCR System (Thermo Fischer Scientific, Waltham, MA, USA). Real-time PCR reactions were performed with 0.5 μL of the RT product in 10 μL-PCR reaction volume, and conducted in triplicate using the 2× SYBR Green PCR Master Mix (Thermo Fischer Scientific, Waltham, MA, USA) and primers purchased from Sigma-Aldrich (St. Louis, MO, USA).

The fold change was calculated by the 2^−ΔΔCt^ method. At least three independent amplifications were performed for each probe evaluated in the triplicate samples.

### 2.4. Immunofluorescence

We used 4% paraformaldehyde (Societa’ Italiana Chimici, Rome, Italy) to fix the cells, and 0.1% *w*/*v* Triton X-100 in PBS for cell permeabilization. Blocking buffer (10% normal goat serum in PBS) was used to incubate cells for 2 h, with primary antibodies against the following: PPARα (Antibodies, Stockholm, Sweden) and Natriuretic Peptide B (BNP) (St John’s Laboratory, London, UK). These were used for the overnight incubation. Subsequently, cells were incubated with Alexa Fluor 555 anti-rabbit secondary antibody (Thermo Fischer Scientific, Waltham, MA, USA), and, after a brief wash, Hoechst 33342 (Thermo Fischer Scientific, Waltham, MA, USA) was used to counterstain the nucleus. F-ACTIN was stained using Phalloidin-Tetramethylrhodamine (Sigma-Aldrich, St. Louis, MO, USA), and nuclei were labelled with Hoechst 33342.

Fluorescence images were acquired using an Olympus Fluoview FV1000 confocal microscope equipped with FV10-ASW version 4.1 software (Olympus Corporation, Tokyo, Japan), using a 40× (1.30 numerical aperture) or a 60× (1.42 numerical aperture) oil objective lens.

### 2.5. Western Blotting

H9c2 cells, cultured in 100 mm dishes-culture below 80% confluence, were lysed in Ripa buffer (50 mmol/L Tris pH 7.5, 150 mmol/L NaCl, 1% *w*/*v* Triton X-100, 1 mmol/L ethylene glycol tetra acetic acid, 1% *w*/*v* sodium deoxycholate), and supplemented with a protease/phosphatase inhibitor cocktail (Thermo Fischer Scientific, Waltham, MA, USA). Total protein content was determined by BCA Protein Assay (Thermo Fischer Scientific, Waltham, MA, USA), and the total cell protein extract was prepared in a Laemmli sample buffer (Biorad, Hercules, CA, USA) and separated by 10% Sodium Dodecyl Sulfate Polyacrylamide Gel Electrophoresis resolving gels. Hybond-C Extra membranes (Thermo Fischer Scientific, Waltham, MA, USA) were used for the electrophoretic proteins transfer. We used 5% *w*/*v* non-fat milk (Cell Signaling Technology, Danvers, MA, USA) in Tris-buffered saline supplemented with Tween 20 at 0.1% *w*/*v* (TBST) for 1 h at room temperature to block the electrophoresis membranes. Immobilized proteins were detected by sequential incubation steps with primary antibodies against the following: PPARα at 1:100 working dilution (GTX101098, GeneTex, Irvine, CA, USA); trimethylation of histone H3 at lysine 27 (H3K27me3) at 1:100 working dilution (C36B11, Cell Signaling Technology, Danvers, MA, USA); acetylation of histone H3 at lysine 27 (H3K27ac) at 1:100 working dilution (PA5-96618, Thermo Fischer Scientific, Waltham, MA, USA); Histone H3 (D1H2) XP^®^ at 1:500 working dilution (4499, Cell Signaling Technology, Danvers, MA, USA); GAPDH (D16H11) XP^®^ at 1:500 working dilution (5174s, Cell Signaling Technology, Danvers, MA, USA); and relative Horseradish peroxidase-conjugated secondary antibody (Jackson ImmunoResearch Labs, Baltimore, PA, USA). Clarity Western enhanced chemiluminescence Substrate (Biorad, Hercules, CA, USA) was used for protein band detection. Image J v3.91 software (open source by the National Institutes of Health) was used for the quantification of protein expression by comparing band intensity.

### 2.6. Cell Proliferation Assay

Cells were plated in a 96-microplate well at a density of 8 × 10^3^ cells/well. Next, 5-bromo-2′deoxyuridine (BrdU) assay was performed by using the Dissociation-Enhanced Lanthanide Fluorescent Immunoassay Cell Proliferation Kit following the manufacturer’s instructions (Revvity, Waltham, MA, USA). Time-resolved fluorometer 2100 Envision^TM^ Multilabel Reader (Perkin Elmer, Waltham, MA, USA) was used to measure cell proliferation as the fluorescent signal is proportional to BrdU incorporation during DNA synthesis.

### 2.7. Cell Viability Assay

Viability was analyzed on 96-well culture plates in which cells were plated at a density of 8 × 10^3^ cells/well, and evaluated by using a commercial XTT (sodium 3′-[1-(phenylaminocarbonyl)-3,4- tetrazolium]-bis (4-methoxy-6-nitro) benzene sulfonic acid hydrate) kit (The Cell Proliferation Kit II, Roche, Indianapolis, IN, USA) according to the manufacturer’s protocol. H9c2 cells were incubated after experimental treatments for 6 h at 37 °C with the XTT labelling mixture. A microplate spectrophotometer Enzyme-linked immunosorbent assay reader (Tecan Austria GmbH, Grödig, Austria) was used to measure the absorbance of the samples.

### 2.8. Flow Cytometer Determination of Apoptosis

The Allophycocyanin Annexin V Apoptosis detection Kit (BD Pharmigen, San Jose, CA, USA) was used to analyze apoptosis. Briefly, cells were washed in PBS and re-suspended in Annexin Binding Buffer (10 mmol/L N-2-hydroxyethylpiperazine-N′-2-ethanesulfonic acid pH 7.4, 140 mmol/L NaCl, and 2.5 mmol/L calcium chloride). Cells were then stained with 0.5 mg/mL Annexin V/7- Amino-Actinomycin D in the dark for 15 min before analysis. The rate of apoptosis was analyzed by flow cytometry within 60 min after the addition of 400 μL of 1× binding buffer to each tube. A Becton Dickinson FACSCanto II flow cytometer (Becton-Dickinson, Milan, Italy, EU) was used for data acquisition and analysis using DiVa Software, version 6.3 (Becton-Dickinson, Milan, Italy, EU).

### 2.9. Assessment of Hypertrophic Phenotype

After various treatments, H9c2 cells were plated at a density of 2 × 10^4^ cells/well in a 4-well chamber slide (Nunc, Naperville, IL, USA), fixed with 4% *w*/*v* paraformaldehyde, and stained with Hematoxylin and Eosin [12]. The light microscope Leica LMD6500 (Leica Microsystem GmbH, Mannheim, Germany), equipped with a digital color camera, was used for the random acquisition of images at 40× magnification. The hypertrophic growth effect of H_2_O_2_ treatment on H9c2 cells was assessed by variations in cell surface area measurement (*n* = 100 per group). Image J v3.91 software (open source by the National Institutes of Health) was used for the quantitative analysis of cell surface area.

### 2.10. Analysis of Reactive Oxygen Species (ROS)

Intracellular ROS content was assessed by 5-(6)-chloromethyl-2′,7′-dichlorodihydrofluorescein diacetate, acetyl ester (CM-H2DCFDA) assay (Thermo Fischer Scientific, Waltham, MA, USA). First, 8 × 10^3^ cells were grown for 24 h in 96 black and flat bottom plates (VIEWPLATE, Perkin Elmer, Waltham, MA, USA), then were incubated for 30 min at 37 °C in 10 μmol/L of CM-H2DCFDA (Thermo Fischer Scientific, Waltham, MA, USA) diluted in KREBS-Henseleit buffer (Sigma-Aldrich, St. Louis, MO, USA). For CM-H2DCFDA detection, the excitation filter and emission filter were set at 495 and 529 nm, respectively. The fluorometric data were then normalized for the cell number by using Hoechst 33342 (Thermo Fischer Scientific, Waltham, MA, USA) and setting the fluorometer filters at 350 nm (excitation wavelength) and 461 nm (emission wavelength). The probe’s fluorescence was monitored with a Synergy H1 Multi-Mode Reader (BioTek^®^ Instruments Inc., Charlotte, VT, USA).

Real-time ROS production changes were also measured using an Incucyte^®^ instrument (Sartorius-Biopharma, Göttingen, Germany).

### 2.11. High Performance Liquid Chromatography Determination of Reduced (GSH) and Oxdized (GSSG) Glutathione

Differently treated cells were sonicated three times for 2 s in 0.1 mL of 0.1 mmol/L potassium-phosphate buffer, pH 7.2 (Sigma-Aldrich, St. Louis, MO, USA). For Free-GSH determinations, 100 μL of 12% sulfosalicylic acid were added to 50 μL of cell lysate, and the acid-soluble free GSH content was determined. For GSSG determination, the cells were sonicated in the presence of 5 mmol/L N-ethylmaleimide (Sigma-Aldrich, St. Louis, MO, USA); 100 μL of 12% *w*/*v* sulfosalicylic acid (Sigma-Aldrich, St. Louis, MO, USA) were added to 50 μL homogenates, and the acid-soluble fraction GSSG content was determined. Results were normalized to protein content assayed by BCA Protein Assay (Thermo Fischer Scientific, Waltham, MA, USA). The derivatization and chromatography procedures were performed, with little modifications, as previously reported [13]. The GSH/GSSG ratio was calculated from reduced GSH and GSSG. Reduced GSH was calculated as follows:

Free-GSH − GSSG = reduced GSH.

### 2.12. Statistical Analysis

All statistical evaluations were performed using GraphPad Prism 8.0 (GraphPad Software, San Diego, CA, USA). The comparison between two groups was performed using a 2-tailed Student’s *t*-test, while for more than two groups a one-way ANOVA test followed by a Fisher LSD Post-Hoc test with Benjamini and Hochberg false discovery rate (FDR) correction was used. A *p*-value < 0.05 was considered statistically significant, whereas a *p*-value < 0.01 and <0.001 was considered highly statistically significant. The measurements are shown as mean ± standard deviation (SD) of three biological replicates repeated at least in technical triplicate.

## 3. Results

### 3.1. Effect of PPARA Silencing on Cell Viability and Apoptosis in Cardiomyoblasts

**Figure 1:**
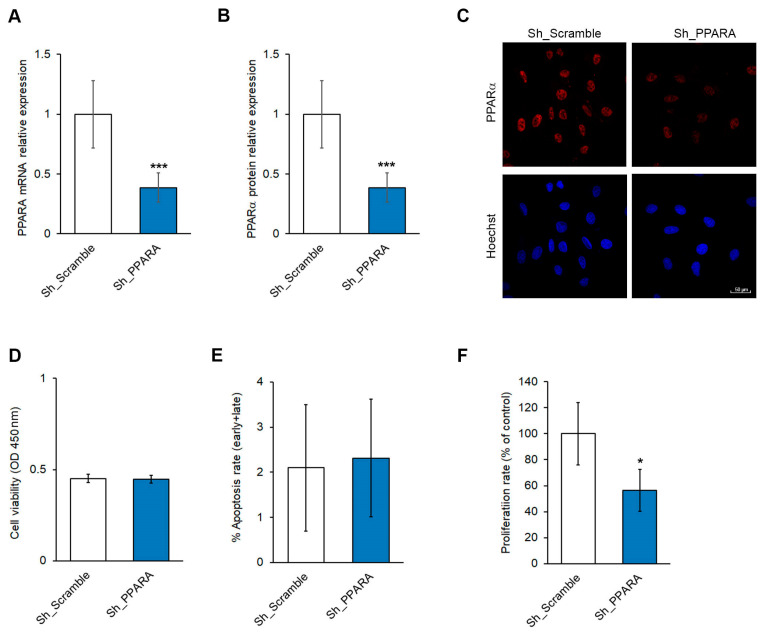
*PPARA* silencing causes a reduction in PPARα gene/protein expression and proliferation rate in H9c2 cells. (**A**) Bar graph of relative mRNA expression of *PPARA* by qRT-PCR; *** *p* < 0.001 vs. Sh_Scramble. (**B**) Bar graph of PPARα protein expression level. GAPDH expression levels were used as a loading reference. *** *p* <  0.001 vs. Sh_Scramble. (**C**) Representative fluorescence images of PPARα protein expression (red color) in Sh_Scramble and Sh_PPARA stable cells. Nuclei were counterstained with Hoechst 33342 (blue color). Scale bar 50 μm. (**D**) Bar graph of cell viability measured by XTT assay. (**E**) The bar graph depicts the percentage of early plus late apoptotic cells measured by flow cytometry. (**F**) Bar graph of cell proliferation rate expressed as a percentage of Europium counts. * *p* <  0.05 vs. Sh_Scramble equal to 100%.

Rat cardiomyoblasts, H9c2, were stably silenced for the expression of the *PPARA* gene. The testing of the silencing model (Sh_PPARA) in comparison to the control (Sh_Scramble) revealed a significant decrease (approximately 50%) in gene and protein expression (Figure 1A,B and Figure S1A). The reduction in PPARα protein expression was also confirmed by immunofluorescence imaging (Figure 1C and Figure S1B). The effect of *PPARA* silencing also caused a downregulation in the transcription of its *ACOX1* target gene (Supplementary Figure S1C), thus supporting a reduction in the PPARα activity. To exclude off-target effects, data were also validated in a *PPARA*-silenced transient model (Supplementary Figure S1D,E) and in rescue experiments (Supplementary Figure S1F,G).

As shown in Figure 1D, stable silencing of *PPARA* was ineffective in affecting cell viability, as measured by XTT. Accordingly, the apoptotic rate (early plus late apoptosis) assessed using flow cytometry remained unchanged in *PPARA* stable-silenced cardiomyoblasts (Figure 1E and Figure S1H). Otherwise, the cell proliferation rate was significantly lower in the Sh_PPARA cells than in control cells (Figure 1F).

### 3.2. Effect of PPARA Silencing on Hypertrophic Phenotype in Cardiomyoblasts

**Figure 2:**
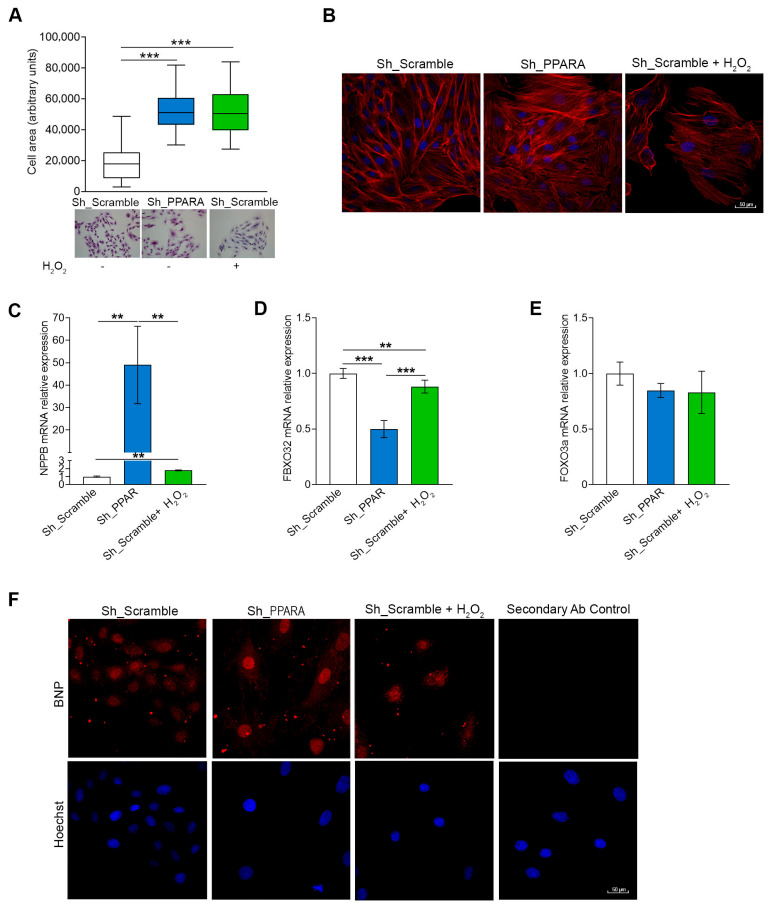
*PPARA* silencing causes cell shape changes, inducing a hypertrophic phenotype in H9c2 cells. (**A**) Box plot (upper panel) of mean cell area (n = 100) using Image J and corresponding representative images (lower panel) of H9c2 cardiomyoblasts morphology by hematoxylin and eosin staining. *** *p* < 0.001 vs. Sh_Scramble. (**B**) Representative images of cytoskeletal structure by merging F-ACTIN labeled with phalloidin (red color) and nuclei stained with Hoechst 33342 (blue color). Scale bar 50 μm. (**C**–**E**) Bar graphs of relative mRNA expression of (**C**) *NPPB*, (**D**) *FBXO32*, and (**E**) *FOXO3a* by qRT-PCR. ** *p* < 0.01; *** *p* <  0.001 vs. Sh_Scramble. (**F**) Representative immunofluorescence images of BNP expression (red color) in Sh_PPARA cells and Sh_Scramble cells with or without H_2_O_2_. Nuclei were counterstained with Hoechst 33342 (blue color). Control of anti-Rb secondary antibody was also reported. Scale bar 50 μm.

To assess the hypertrophic phenotype, we analyzed cell shape by hematoxylin and phalloidin staining. As shown in Figure 2A and Figure S2, the *PPARA* gene silencing caused an enlargement of cell area with respect to Sh_Scramble cells, inducing a hypertrophic phenotype similar to that observed in Sh_Scramble cells treated for 1 h with 50 μM H_2_O_2_. Moreover, we observed a strong effect on the cytoskeletal structure in both Sh_Scramble + H_2_O_2_ and Sh_PPARA cells in comparison to untreated Sh_Scramble cells (Figure 2B). In particular, we found a noticeable reordering of the actin cytoskeleton in which the number of actin bundles is higher and their distance smaller, indicating a region of greatest stiffness in Sh_PPARA.

Then, we assessed the mRNA expression of some hypertrophic genes, such as the *NPPB* gene encoding for Protein B-type natriuretic peptide (BNP), the *FBXO32* gene encoding for Atrogin-1, and FOXO3a encoding for the Forkhead box O3a transcription factor. As reported in Figure 2C,D, *NPPB* gene expression was significantly upregulated, while *FBXO32* transcript was significantly downregulated in Sh_PPARA cells compared to Sh_Scramble cells. Otherwise, *PPARA* silencing was ineffective on *FOXO3* transcription (Figure 2E). In order to verify the BNP protein expression increase, we performed immunofluorescence experiments (Figure 2F). All these results indicate that *PPARA*-silenced cells mimic a hypertrophic cardiac model.

### 3.3. Effect of PPARA Silencing on Redox Metabolism and Ferroptosis in Cardiomyoblasts

**Figure 3:**
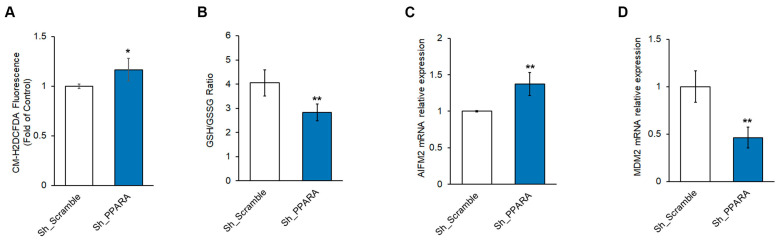
PPARA silencing affects the oxidative response and ferroptosis genes in H9c2 cells. (**A**) Bar graph of ROS production by measuring the CM-H2DCFDA fluorescence intensity expressed as fold change with respect to Sh_Scramble equal to 1. * *p* < 0.05. (**B**) Bar graph of GSH/GSSG ratio. ** *p* < 0.01 vs. Sh_Scramble. (**C**,**D**) Bar graph of relative mRNA expression of (**C**) *AIFM2* and (**D**) *MDM2* by qRT-PCR. ** *p* < 0.01 vs. Sh_Scramble.

We evaluated the effect of *PPARA* stable silencing on redox metabolism in H9c2 cells by different assays. The analysis of ROS (Figure 3A and Figure S3A) showed a significant increase in levels of these oxidative markers in Sh_PPARA stable cells with respect to Sh_Scramble cells. The assessment of cell redox status by the GSH/GSSG ratio revealed that the silenced Sh_PPARA stable cells displayed a significant decrease of this ratio when compared to Sh_Scramble cells (Figure 3B and Figure S3B,C).

As we found a redox unbalance in cells silenced for *PPARA*, and since ROS accumulation might contribute to ferroptosis switching, we verified the PPARα role in the ferroptosis pathway by assessing the mRNA expression levels of some genes involved in this process, including AIF family member 2 (*AIFM2*), mouse double minute 2 homolog (*MDM2*), glutathione peroxidase 4 (*GPX4*), and *NFE2*. We observed a significant increase in *AIFM2* transcription and a significant decrease in *MDM2* transcription in Sh_PPARA cells with respect to Sh_Scramble cells (Figure 3C,D). We also observed a reduced trend of *NFE2* expression, and no changes in *GPX4* mRNA levels in Sh_PPARA cells with respect to Sh_Scramble (Supplementary Figure S3E,F).

### 3.4. Effect of PPARA Silencing on Epigenetic Mechanisms Upstream of BNP Expression in Cardiomyoblasts

**Figure 4:**
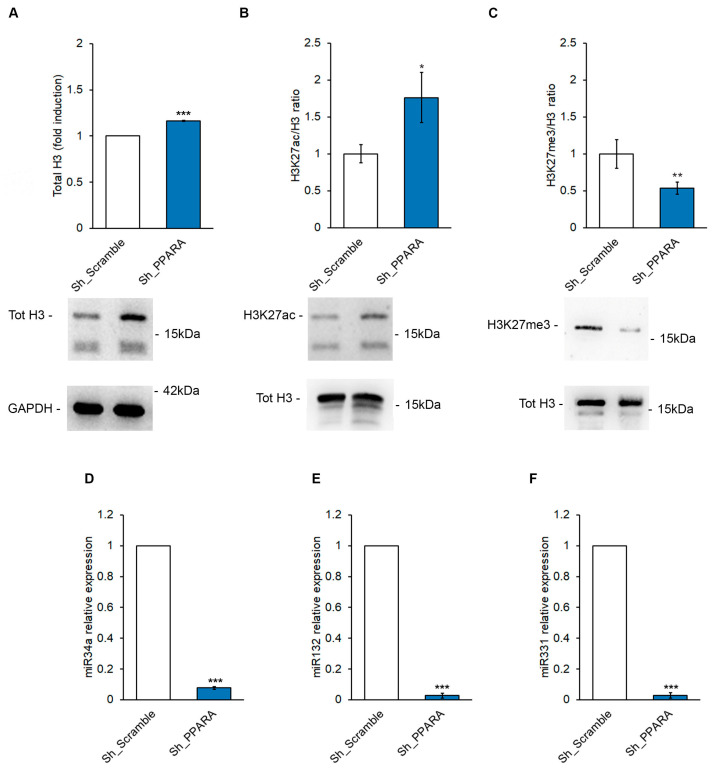
*PPARA* silencing determines epigenetic modulation in correspondence with Histone H3 in H9c2 cells. Quantification (upper panels) and representative immunoblotting (lower panels) of the protein expression of (**A**) total histone H3, (**B**) H3K27ac, and (**C**) H3K27me3 in Sh_Scramble and Sh_PPPARA cells. GAPDH expression levels were used as a loading reference for total H3 quantification, while the evaluation of H3K27ac and H3K27me3 levels was assessed as a ratio with respect to total H3. * *p* <  0.05; ** *p* <  0.01; *** *p* <  0.001 vs. Sh_Scramble. Bar graph of relative miRNA detection of (**D**) rno-miR-34a, (**E**) rno-miR-132, and (**F**) rno-miR-331 by qRT-PCR. *** *p* <  0.001 vs. Sh_Scramble.

The effects of PPAR silencing on the transcription of the *NPPB* gene could be explained by the different epigenetic mechanisms of its promoter regulation [14,15]. Wei and collaborators [16] reported that pressure overload-induced murine cardiac hypertrophy regions near the *NPPB* promoter are enriched in histone H3 acetylation, which is associated with a recruitment of the p300 protein, thus inducing the transcriptional activation of the *NPPB* gene. Our data demonstrated that *PPARA* silencing may lead to an increased expression of total histone H3 and H3K27ac (Figure 4A,B), and a decrease in H3K27me3 levels (Figure 4C), thereby supporting the hypothesis that these two histone modifications may contribute to increased *NPPB* gene transcription.

An additional epigenetic mechanism involved in regulating the *NPPB* transcript was mediated by miRNAs [15]. Therefore, we performed a search using TargetScan software, release 8 (accessed on July 25, 2025) [17], which revealed only 13 poorly conserved miRNAs that could target the 3′ untranslated region (3′UTR) of the *NPPB* promoter (Table S2). Among these, we validated miR34a, miR132, and miR331, which previous studies have reported as upregulated in cardiac hypertrophy [18,19,20]. Our results showed a statistically significant decrease in all these miRNAs in Sh_PPARA-silenced cells compared to the control (Sh_Scramble) (Figure 4D–F), thus reinforcing the hypothesis of PPARα-mediated epigenetic control of *NPPB* transcription.

### 3.5. Assessment of Hypertrophic Phenotype and Oxidative Stress After Ebs Treatment in Hypertrophic Cardiomyoblasts

**Figure 5:**
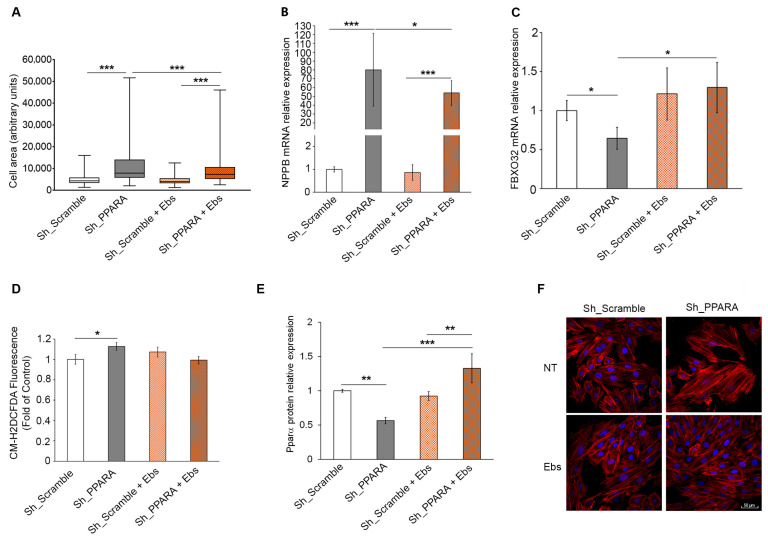
Rescue effect of Ebs on redox metabolism in *PPARA*-silenced H9c2 cells. (**A**) Box plot of mean cell area (n = 100) by using Image J. (**B**,**C**) Bar graphs of relative mRNA expression of *NPPB* and *FBXO32* mRNA expression level by qRT-PCR. (**D**) Bar graph of ROS production by measuring the CM-H2DCFDA fluorescence intensity expressed as fold change with respect to Sh_Scramble equal to 1. (**E**) Quantitative analysis of PPARα protein expression level. GAPDH expression levels were used as loading reference. *** *p* <  0.001; ** *p* < 0.01; * *p* < 0.05. (**F**) Representative images of cytoskeletal structure, F-actin labeled with phalloidin. Red: phalloidin; nuclei are counterstained with Hoechst 33342 (blue color), and combined images are shown. Scale bar 50 μm.

Since our *PPARA* depletion-dependent hypertrophic phenotype was characterized by redox imbalance, we evaluated the possible effect of Ebs, a synthetic organoselenium molecule acting as a mimic of GPX that possesses antioxidant, anti-ferroptosis, and anti-inflammatory properties [10,11]. In our experimental conditions, we observed a reduction in cell dimensions after Ebs treatment in *PPARA*-silenced cells (Figure 5A) without significant changes in cell viability (Supplementary Figure S4A). This was confirmed by a corresponding decrease in *NPPB* mRNA expression in *PPARA*-silenced cells, while the *FBXO32* mRNA expression increased (Figure 5B,C). Furthermore, we obtained a slight but statistically significant decrease in ROS production (Figure 5D). These results could be explained by a significant Ebs-dependent increase in residual PPARα protein expression (Figure 5E and Figure S4B). Moreover, Ebs treatment also ameliorated the reordering of the cytoskeleton, indicating a reduced stiffness in Sh_PPARA cells, restoring a phenotype similar to Sh_Scramble cells (Figure 5F).

### 3.6. Assessment of Epigenetic Changes After Ebs Treatment in Hypertrophic Cardiomyoblasts

**Figure 6:**
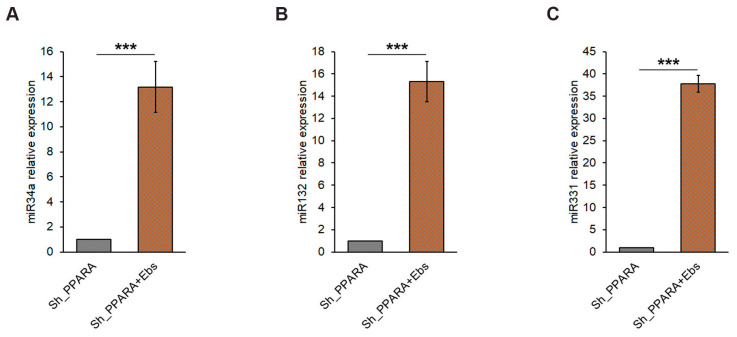
Rescue effect of Ebs on miRNAs levels in *PPARA*-silenced H9c2 cells. Bar graphs of relative miRNAs detection of (**A**) rno-miR-34a, (**B**) rno-miR132, and (**C**) rno-miR331 by qRT-PCR. miRNAs levels are expressed as fold change with respect to Sh_PPARA-silenced cells equal to 1. *** *p* < 0.001.

The restoration of epigenetic changes observed in *PPARA*-silenced cells could be a mechanism explaining the Ebs-dependent partial recovery of a normal phenotype in hypertrophic cardiomyoblasts. In particular, the Ebs treatment per se and in PPARA-depleted cells increased H3K27me3 levels (Supplementary Figure S5A), while it seems to have no effect on H3K27ac levels (Supplementary Figure S5B). Moreover, when the Ebs treatment was tested for the evaluation of effects on miRNAs affected by *PPARA* silencing, we observed a very strong rescue of the expression of miR34A (Figure 6A), miR132 (Figure 6B), and miR331 (Figure 6C).

## 4. Discussion

PPARα is a ligand-activated transcription factor that is the master regulator gene involved in the lipid metabolism of various tissues. PPARα may play a key role in transactivating or repressing gene expression in a DNA-binding-independent manner by interfering with other vital players implicated in signaling pathways, thereby regulating various processes such as metabolism, inflammation, proliferation, and differentiation. If deregulated, these processes are associated with a broad range of human diseases [21]. Numerous reports have indicated that the *PPARA* gene regulatory pathway influences cardiovascular diseases through its role in metabolic regulation. Reduced PPARα expression and excessive mitochondrial fatty acid oxidation contribute to alterations such as heart failure, ischemic heart disease, and diabetic cardiomyopathy [22,23,24]. Additionally, PPARα overexpression reduces doxorubicin-induced cardiotoxicity by inhibiting mitochondria-dependent apoptosis [23]. It is reported that PPARα regulates the balance of redox responses in cardiac tissue and during aging [24]. Indeed, *PPARA*-KO mice increase cardiac pyruvate dehydrogenase flux as a compensatory mechanism, perhaps to maximize ATP production, indicating an age-dependent reliance on glucose metabolism in *PPARA*-KO mice [25].

In this study, we uncovered the role of *PPARA*-silencing on hypertrophic phenotype in H9c2 rat cardiomyoblasts. Although the embryonic nature of these cells suggests caution in their use as cardiomyocytes, they represent, in their undifferentiated status, a widely accepted model to investigate phenotypic changes occurring during hypertrophic stimuli, as revealed also by our morphological results [9]. We validated the in vitro model at the molecular and biochemical levels of PPARα depletion, and by expression levels of some hypertrophic markers such as *NPPB* and *FBXO32*, which were increased and decreased, respectively, according to hypertrophic conditions [14,26]. BNP is considered a sensitive diagnostic biomarker for heart failure onset and disease progression [27]. In particular, BNP and N-terminal proBNP, the cleavage product generated by proBNP processing to mature BNP, are increased in patients [14]. *FBXO32* provides a regulatory mechanism to balance the process, causing CH both in vivo and *in vitro*. Indeed, *FBXO32* inhibits pathologic CH by participating in a ubiquitin ligase complex that targets calcineurin for proteasome-dependent degradation, a factor involved in causing pathologic hypertrophy [26,28].

In our study, *PPARA* silencing did not affect cell viability and apoptosis susceptibility. Indeed, it has been reported that apoptosis susceptibility is associated with increased PPARα expression [29,30]. Moreover, we found a statistically significant reduction in cell proliferation rate in *PPARA*-silenced cells that could be explained by the presence of hypertrophic cells that sometimes appear polynucleated. It is reported that cardiomyocytes undergo a cell cycle modulation from proliferation to the hypertrophy process in the post-natal heart [31], known as maturational hypertrophy, in which the cardiomyocyte volume increases despite the cell cycle withdrawal. In order to explain the proliferation rate of cardiomyocytes after a heart injury, there are two hypotheses: the presence of binucleated cells or the increase in cellular size. Accordingly, in our in vitro model, *PPARA*-silenced cells were characterized by enlarged cellular area (hematoxylin and eosin staining) and by actin-F filaments aggregated in disorganized bundles (phalloidin staining). The same morphological and biomolecular (i.e., *NPPB* and *FBXO32*) hypertrophic phenotype characteristics were obtained in the control cell line only after H_2_O_2_ treatment.

However, PPARα is the master regulator gene for lipid homeostasis by balancing the expression of genes involved in fatty acid transport and oxidation. PPARα modulates the expression of essential genes for mitochondrial fatty acid β-oxidation, such as *ACOX1* [17]. Accordingly, in *PPARA*-silenced cells, we found a significant decrease in *ACOX1* mRNA expression level with respect to the control cells. As the *ACOX1* gene encodes the first enzyme of the fatty acid β-oxidation pathway, the probable consequence of this decrease could be the metabolic switch toward glucose consumption, with an increase in the glycolysis pathway. This aspect could be further investigated in our model.

As Guellich and collaborators [5] demonstrated the role of oxidative stress in the cardiac dysfunction of *PPARA*^-/-^ mice, here we evaluated the effect of *PPARA* silencing on oxidative stress. ROS homeostasis depends on the balance between the antioxidant systems and redox signaling, contributing to cell viability under stress stimuli [32]. In *PPARA*-silenced cells, we reported a slight, but significant, increase in ROS production with respect to the control cells. It is reported that many transcriptional factors and proteins involved in hypertrophic processes are subjected to redox regulation following the glutathionylation of susceptible cysteine residues [13]. GSH is usually present in its reduced form, and is converted into its oxidized form by stimulation such as oxidative stress. The GSH/GSSG ratio is thus a valuable indicator of oxidative stress in tissue and cells [33]. In our study, we show a decrease in this ratio, indicating an alteration of oxidative balance in *PPARA*-silenced cells with respect to the control. This imbalance might be responsible for iron-dependent lipid peroxidation, driving towards a form of regulated cell death known as ferroptosis [34]. In order to investigate the PPARα role in ferroptosis, we assayed the mRNA expression level of some possible ferroptosis-related genes, including *GPX4*, *AIFM2*, *MDM2*, and *NFE2*, in our models. Even though, under *PPARA* silencing, non-significant effects were observed in *GPX4* and *NFE2* mRNA levels, the depletion of PPARα significantly increased *AIFM2* and decreased *MDM2* transcription levels. Accordingly, the *AIFM2* pathway was reported as one of the mitochondrial pathways linked to ferroptosis in cardiac diseases [35]. The observed reduction in *MDM2* transcription is in line with previous evidence where Hauck et al. [36] demonstrated that the cardiac depletion of *MDM2* in mice caused a concentric cardiac hypertrophy strongly associated with the downregulation of the PPARα pathway and an increase in ROS. Furthermore, Xing et al. [37] also reported that *PPARA*^−/−^ mice displayed higher ROS levels, lower levels of hepatic GSH, and were more sensitive to ferroptosis when fed with a high-iron diet.

An explanation of the hypertrophic phenotype characterized by *NPPB* upregulation observed in *PPARA*-silenced cells could be a possible epigenetic modulation mediated by the dynamic regulation of H3K27 in its promoter region [15]. We found a statistically significant increase in H3K27ac and a decrease in H3K27me3 expression in our *PPARA*-silenced cells. Epigenetic modifications, such as histone acetylation and methylation, are associated with adult CH and heart failure. Indeed, genome-wide analysis in heart hypertrophy has evidenced many genes and their enhancers modified through histone-3 lysine-27 acetylation, associated with gene activation, and histone-3 lysine-27 trimethylation, associated with gene repression and compacted heterochromatin [38]. In particular, *NPPB* reactivation related to epigenetic modifications at its promoter region was reported in patients with heart failure [38].

MiRNAs represent a further epigenetic regulatory mechanism upstream of *NPPB* transcription [15]. Multiple miRNAs modulate the expression of the *NPPB* mRNA in cardiomyocytes by binding to its 3′-UTR in a sequence-specific manner. TargetScan provided us with a list of 13 miRNAs with only poorly conserved sites on the *NPPB* promoter in the rat. Among these, we investigated three specific miRNAs, including miR34a, miR132, and miR331, that were also previously associated with cardiac hypertrophy [15,18,19,20]. Although our study lacks a confirmation of direct mechanisms that may link these three miRNAs to the *NPPB* promoter (e.g., luciferase assays and mimic effects), we found that the hypertrophic model caused by PPARα depletion exhibited a significant downregulation of all these miRNAs. Conversely, most of the evidence reported these miRNAs as potential therapeutic targets in cardiac hypertrophy and remodeling [39,40]. Although these findings highlight that further studies are necessary to clarify the epigenetic involvement in cardiac hypertrophy, they suggest that the use of compounds able to interfere with epigenetic mechanisms could be a helpful approach. In particular, besides its antioxidant properties [11], Ebs could be considered a pan-epigenetic inhibitor due to its targeting of erasers and readers [41,42]. As expected, in our experimental condition, Ebs treatment reduced hypertrophic phenotype and oxidative stress, and determined an upregulation of H3K27me3 levels and miRNAs expression in PPARα-silenced cells, thus restoring a condition that resembled the normal cardiomyoblasts. Particularly, the latter epigenetic effects of Ebs merit further investigation.

In conclusion, our data highlight that PPAR silencing triggers morphological and molecular changes that prompt hypertrophy in rat cardiomyoblasts, and that antioxidant and anti-ferroptosis Ebs treatment can revert these modifications, thus opening a new scenario for deep pre-clinical studies on the therapeutic value of this small molecule in human CH.

## Data Availability

The data that support the findings of this study are available from the corresponding authors upon reasonable request.

## Supplementary Materials

The following supporting information can be downloaded at https://www.mdpi.com/article/10.3390/antiox14091059/s1: Supplementary Methods; Table S1: Primer list; Figure S1: In vitro model of *PPARA*-silenced H9c2 cells; Figure S2: High-resolution images of the lower panels reported in Figure 2A; Figure S3: Oxidative stress and ferroptosis in *PPARA*-silenced H9c2 cells; Table S2: Characteristics of the 13 poorly conserved miRNAs that could target the 3′UTR region of the *NPPB* promoter; Figure S4: Effects of Ebs on stable *PPARA*-silenced H9c2 cells; Figure S5: Effect of Ebs on H3K27ac and H3K27me3 in *PPARA*-silenced H9c2 cells.

## Abbreviations

The following abbreviations are used in this manuscript:

3′UTR	3′-untranslated region
ACOX1	Acyl-coenzyme A oxidase 1 gene
AIFM2	AIF family member 2
BNP	Protein B-type natriuretic peptide
CH	Cardiac hypertrophy
CM-H2DCFDA	5-(6)-Chloromethyl-2′,7′-dichlorodihydrofluorescein diacetate, acetyl ester
Ebs	Ebselen
FBXO32	Muscle Atrophy F-box gene
FOXO3	Forkhead box O3 gene
GAPDH	Glyceraldehyde-3-Phosphate Dehydrogenase gene
GSH	Reduced glutathione
GSSG	Oxidized glutathione
H_2_O_2_	Hydrogen peroxide
MDM2	Mouse double minute 2 homolog
NFE2	Nuclear factor erythroid 2 gene
NPPB	Brain Natriuretic Peptide B gene
PBS	Phosphate Buffered Saline
PPAR	Peroxisome proliferator-activated receptor family of lipid-activated nuclear receptors
ROS	Reactive oxygen species
SYBR-green	2-[N-(3-dimethylaminopropyl)-N-propylamino]-4-[2,3-dihydro-3-methyl-(benzo-1,3-thiazol-2-yl)-methylidene]-1-phenyl-quinolinium
