# Supplementary material for: Peroxisome Proliferator-Activated Receptor Family of Lipid-Activated Nuclear Receptors Alpha Silencing Promotes Oxidative Stress and Hypertrophic Phenotype in Rat Cardiac Cells
Source: Antioxidants (Basel). 2025 Aug 28;14(9):1059. doi: 10.3390/antiox14091059 (PMC12466375; doi:10.3390/antiox14091059)
Supplement: Supplementary file 1 [file antioxidants-14-01059-s001.zip › antioxidants-3822946-supplementary.pdf]

Supplementary Data

Peroxisome Proliferator-Activated Receptor Family of Lipid-Activated Nuclear Receptors Alpha Silencing Promotes Oxidative Stress and Hypertrophic Phenotype in Rat Cardiac Cells

Marzia Bianchi [1], Nadia Panera [1], Sara Petrillo [2], Nicolò Cicolani [3], Cristiano De Stefanis [3], Marco Scarsella [3], Domenico Ciavardelli [4], Fiorella Piemonte [2], Anna Alisi [1,*] and Anna Pastore [1]

[1]Research Unit of Genetics of Complex Phenotypes, Bambino Gesù Children's Hospital, IRCCS, Rome, Italy; marzia.bianchi@opbg.net (M.B.); nadia.panera@opbg.net (N.P.); anna.alisi@opbg.net (A.A); anna.pastore@opbg.net (A.P.)

[2]Unit of Muscular and Neurodegenerative Diseases, Bambino Gesù Children's Hospital, IRCCS, Rome, Italy; sara.petrillo@opbg.net (S.P.); fiorella.piemonte@opbg.net (F.P.)

[3]Core Facilities, Bambino Gesù Children's Hospital, IRCCS, Rome, Italy; nicolo.cicolani@opbg.net (N.C.); cristiano.destefanis@opbg.net (C.D.S.); marco.scarsella@opbg.net (M.S.)

[4]School of Medicine and Surgery, University "Kore" of Enna, Italy. domenico.ciavardelli@unikore.it

*Correspondence: anna.alisi@opbg.net; Tel.: +390668592186

List of Tables and Figures:

Table S1 page 2
Figure S1 page 3
Figure S2 page 4
Figure S3 page 5
Table S2 page 6
Figure S4 page 7
Figure S5 page 7

Table S1. Primers list.

AIFM2 (Rn)-F	5′-CCTTGCCCTTCTCACATCTTAT-3′
AIFM2 (Rn)-R	5′-CTGCTTCACCATGTCCTCATAG-3′
MDM2 (Rn)-F	5′-GGTTGTGGGCTGCAGAGAAG-3′
MDM2 (Rn)-R	5′-TGCAAGGATCAAATGTGTGTCA-3′
GPX4(Rn)-F	5′-CCGTCTGAGCCGCTTATTGAA-3′
GPX4(Rn)-R	5′-ACACGCAACCCCTGTACTTA-3′
GAPDH (Rn)- F	5′-TCTCTGCTCCTCCCTGTTCTA-3′
GAPDH(Rn)- R	5′-GGTAACCAGGCGTCCGATAC-3′
rno-miR-34a-F	5′-TGGCAGTGTCTTAGCTGGTTG-3′
rno-miR-34a-R	5′-GGCAGTATACTTGCTGATTGCTT-3′
rno-miR-132-F	5′-AACCGTGGCTTTCGATTGTTA-3′
rno-miR-132-R	5′-CGACCATGGCTGTAGACTGTTAC-3′
rno-miR 331-F	5′-GGTCTTGTTTGGGTTTGTT-3′
rno-miR 331-R	5′-GAACATGTCTGCGTATCTC-3′
rno-U6-F	5′-TCG CTT CGG CAG CAC ATA TAC-3′
rno-U6-R	5′-TAT GGA ACG CTT CAC GAA TTT G-3′

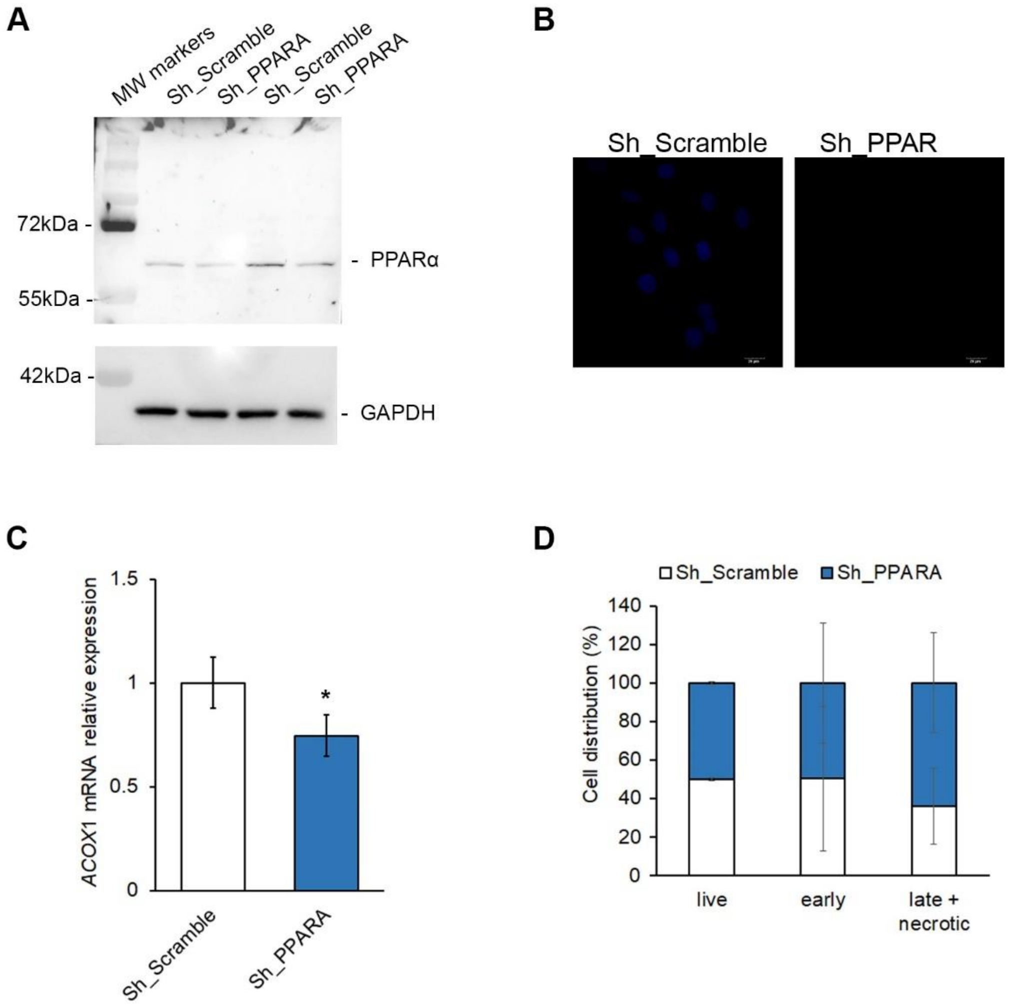

Figure S1. In vitro model of PPARA-silenced H9C2 cells. (**A**) Representative original immunoblotting of PPARα expression. GAPDH was acquired as a loading reference. (**B**) Control of secondary antibody anti-rabbit Alexa Fluor 555 for the panel of images in Figure 1C. (**C**) Bar graph of relative mRNA expression of *ACOX1* by qRT-PCR; *p< 0.05 vs Sh_Scramble. (**D**) Bar graph of flow cytometric analysis of apoptotic cell population distribution after staining with Annexin V detection kit.

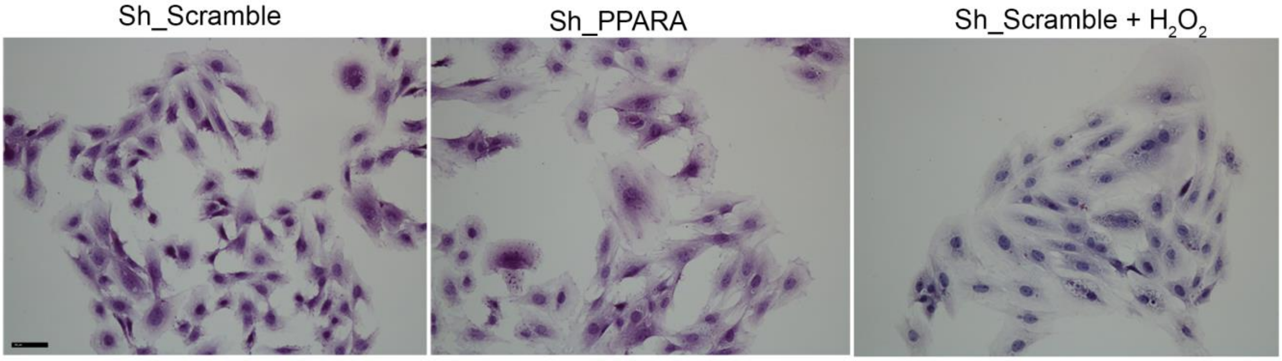

Figure S2. High-resolution images of the lower panels reported in Figure 2A.

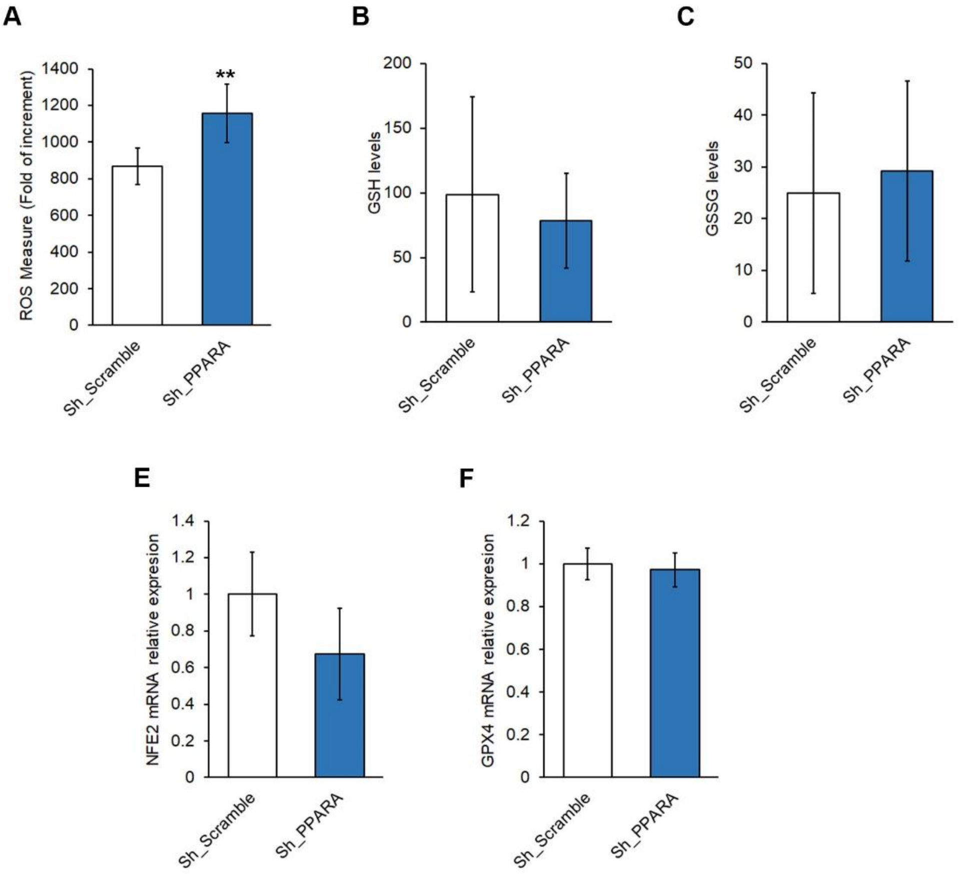

Figure S3. Oxidative stress and ferroptosis in PPARA-silenced H9C2 cells. (**A**) Bar graph of ROS production by measuring the CM-H2DCFDA analyzed by Incucyte system. **p< 0.01; vs Sh_Scramble. (**B-C**) Bar graph of the levels of (**B**) GSH, and (**C**) GSSG. **(D-E**) Bar graph of relative mRNA expression of (**E**) *NFE2*, and (**F**) *GPX4* by qRT-PCR. **p< 0.01; vs Sh_Scramble.

Table S2. Characteristics of the 13 poorly conserved miRNAs that could target the 3′UTR region of the NPPB promoter.

miRNA	Position in the UTR	seed match	Context ++ score	Context ++ score percentile	weighted context ++ score	conserved branch length	Pct	Predicted relative KD
rno-miR-203b-5p	66-72	7mer-m8	-0.33	90	-0.33	0	N/A	N/A
rno-miR-323-5p	67-73	7mer-m8	-0.36	95	-0.36	0.113	N/A	N/A
rno-miR-34a-3p	122-128	7mer-m8	-0.47	98	-0.47	0	N/A	N/A
rno-miR-331-5p	134-140	7mer-m8	-0.32	96	-0.32	0.113	N/A	N/A
rno-miR-132-3p	138-144	7mer-m8	-0.42	99	-0.42	0.375	< 0.1	N/A
rno-miR-212-3p	138-144	7mer-m8	-0.43	99	-0.43	0.375	< 0.1	N/A
rno-miR-7b	150-156	7mer-1A	-0.19	91	-0.19	0	< 0.1	N/A
rno-miR-7a-5p	150-156	7mer-1A	-0.19	91	-0.19	0	< 0.1	N/A
rno-miR-218a-5p	156-162	7mer-1A	-0.22	91	-0.22	1.705	< 0.1	N/A
rno-miR-31a-5p	164-170	7mer-1A	-0.37	97	-0.37	0.113	< 0.1	N/A
rno-miR-3587	171-177	7mer-1A	-0.36	98	-0.36	0	N/A	N/A
rno-miR-3572	172-178	7mer-1A	-0.25	95	-0.25	0	N/A	N/A
rno-miR-3099	185-191	7mer-1A	-0.38	97	-0.22	0	N/A	N/A

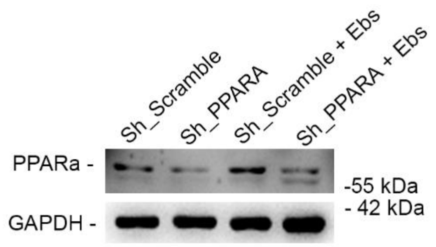

Figure S4. Representative immunoblotting of PPARα quantification reported in Figure 5E.

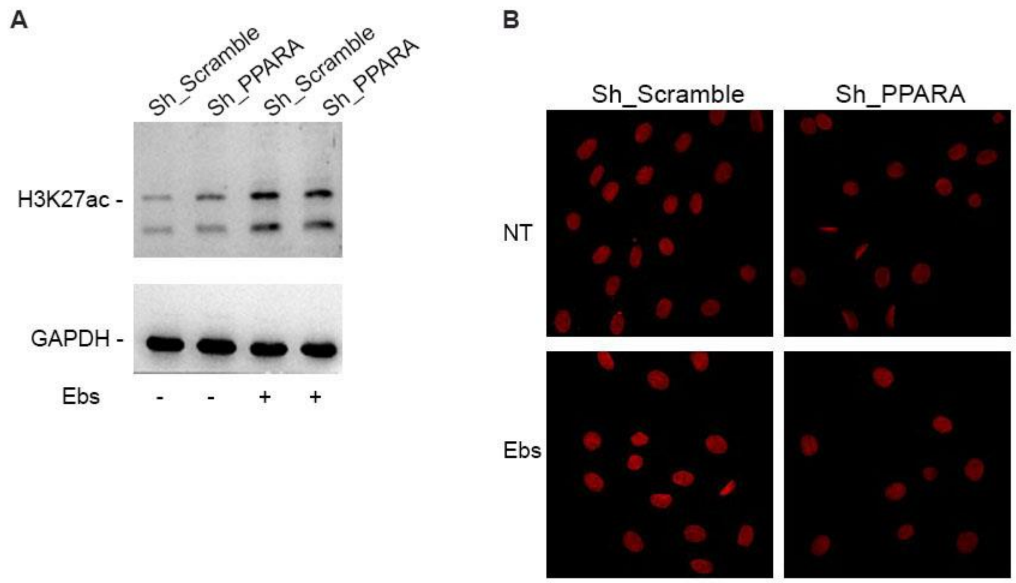

Figure S5. Effect of Ebs on H3K27ac and H3K27me3 in PPARA-silenced H9c2 cells. (**A**) Representative immunoblotting of H3K27ac. (**B**) Representative immunofluorescence of H3K27me3. Magnification 60x.
